# Ultrashort pulse laser ablation in liquids: probing the first nanoseconds of underwater phase explosion

**DOI:** 10.1038/s41377-022-00800-0

**Published:** 2022-04-27

**Authors:** Chaobo Chen, Leonid V. Zhigilei

**Affiliations:** Department of Materials Science and Engineering, University of Virginia, 395 McCormick Road, Charlottesville, VA 22904-4745 USA

**Keywords:** Laser-produced plasmas, Imaging and sensing

## Abstract

The ultrafast pump-probe microscopy has shed new light on the complex dynamics of laser-induced explosive phase transformations and highlighted the importance of close integration of experimental, computational, and theoretical efforts.

## Graphical abstract

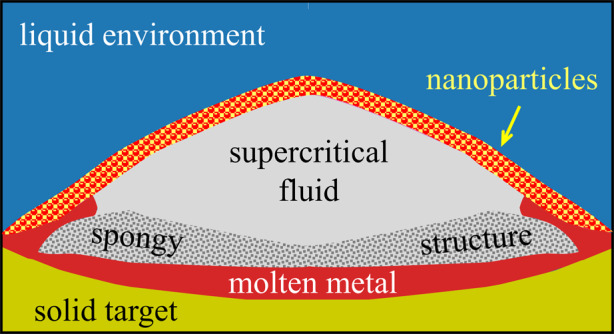

The ultrashort pulse laser ablation in liquids is a phenomenon of high practical importance, with applications ranging from surface nanostructuring^1^ to synthesis of chemically clean colloidal nanoparticles of high demand in the fields of biomedicine and catalysis^2,3^. Yet, despite the practical relevance, the fundamental understanding of laser ablation in liquids remains far from being complete. The relatively slow progress in this area is largely related to the highly nonequilibrium and inherently multiscale nature of processes responsible for the material removal from the irradiated target and its transformation into a colloidal solution of nanoparticles. The laser ablation in air or vacuum is already a complex phenomenon, which involves a strong electronic excitation, energy redistribution in the electronically excited state, electron-phonon equilibration, rapid heating and melting, ultrafast mechanical deformation and photomechanical spallation, superheating of the molten material and its explosive decomposition (“phase explosion”) into vapor and liquid droplets^4^. The presence of a liquid environment adds significantly to the complexity of the ablation phenomenon, as the hot products of the explosive phase decomposition interact dynamically with the liquid and trigger the formation and expansion of a cavitation bubble.

Imaging of the cavitation bubbles, usually performed with time-resolved shadowgraphy^5,6^, combined with laser-scattering^7^, or X-ray probing^8,9^ of the interior of the cavitation bubbles, has provided valuable information on the expansion and collapse of the cavitation bubbles, as well as the evolution of the size and crystallinity of the nanoparticles. The initial stage of the ablation process at the origin of the emergence of the cavitation bubble and the nanoparticles, however, has largely remained beyond reach for in situ experimental probing. In studies of laser ablation in vacuum or air, the optical pump-probe imaging of surface reflectivity has been successful in revealing the phase transformations associated with large reflectivity changes or generation of transient structures producing characteristic optical interference signatures, such as Newton rings^10,11^. The added complexity of the ablation in liquids, however, creates a significant challenge for the interpretation of multi-stage variation of the reflectivity signal.

In a recent study of laser ablation of Au in water reported by Spellauge et al.^12^, the challenge of interpretation of the results of time- and spatially-resolved optical imaging is met by making direct links to the predictions of large-scale atomistic modeling^6,13–15^. Using the time-resolved reflectivity measurements performed across all relevant timescales, Spellauge et al. are able to reconstruct a complete dynamic picture of the ablation process, from the electronic excitation and injection of electrons into the water environment during the first picoseconds after the excitation, to the dynamic interaction of the ablation plume with water and the emergence of a cavitation bubble on a nanosecond timescale, and to the expansion and collapse of the cavitation bubble on the scale of tens of microseconds. The measurements are found to be consistent with computational predictions of the explosive phase decomposition of a surface region of the irradiated target, rapid deceleration of the ablation plume by the water environment, accumulation of a hot metal layer at the plume-water interface, and formation of nanoparticles in the plume-water mixing region^13^. The experimental confirmation of the prompt formation of nanoparticles at the plume-water interface within the first nanoseconds of the ablation process is particularly remarkable, as it goes against the common assumption of the nanoparticle formation at a much later stage, inside the expanding cavitation bubble.

The interpretation of the reflectivity measurements, however, is far from being straightforward and involves a number of assumptions that still need to be verified in future targeted experiments and simulations. The theoretical description of the transient optical properties of a material undergoing highly nonequilibrium phase transformations is challenging and calls for the development of advanced computational methods. Moreover, the time-resolved optical imaging is only providing two-dimensional (2D) maps of the three-dimensional (3D) ablation process. The variation of material density, temperature, and phase state in the direction normal to the irradiated surface is illustrated in Fig. 1 by a series of snapshots from large-scale atomistic simulations of laser ablation of an alloy FeNi target irradiated by a 10 ps laser pulse in vacuum and water. The thermodynamic maps in Fig. 1c, d reveal the layered structure of the emerging ablation plume. In the case of ablation in water, the ablation plume consists of a plume-water interfacial layer with conditions suitable for the nucleation and growth of atomic clusters and nanoparticles, a layer of metal brought to the supercritical state, a layer of spongy structure generated by the unloading of laser-induced pressure, a spalled layer of molten metal topping the spongy structure at the periphery of the laser spot, and a layer of the molten pool. The translation of such 3D sandwiched structures into 2D maps of reflectivity not only presents a significant challenge but also leads to an unavoidable loss of critical information about the ablation process.

**Fig. 1:**
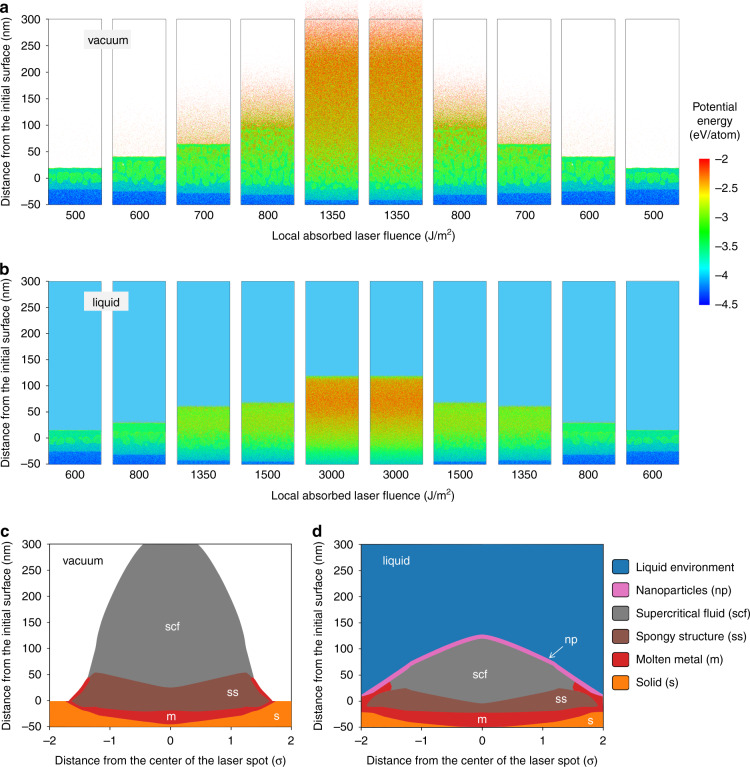
The initial stage of laser ablation predicted in atomistic simulations. The results of large-scale atomistic simulations are shown for FeNi alloy targets irradiated by 10 ps laser pulses in vacuum (**a**, **c**) and in water (**b**, **d**). The snapshots from simulations performed at different values of local laser fluence realized in different parts of a laser spot are shown in (**a**, **b**) for a time of 100 ps after the laser pulse. The atoms in the snapshots are colored by potential energy, with blue, green, and red colors corresponding to the solid, liquid, and vapor phases. The corresponding maps of the phase state of the material in different parts of the ablation plumes generated by laser beams with Gaussian spatial profiles with standard deviation σ and peak absorbed laser fluences of 1500 J/m^2^ and 3000 J/m^2^ are shown in (**c**) and (**d**), respectively. The phase maps are based on the analysis of density and temperature distributions predicted in the atomistic simulations. Note that the images are shown with a large aspect ratio between the lateral and vertical dimensions to provide a clear view of distinct layers of the ablation plume, which remain essentially flat at this early stage of the ablation process

Nevertheless, the demonstrated ability of ultrafast pump-probe microscopy to yield insights into the early-stage ablation dynamics^12^ is encouraging and suggests that further progress can be achieved through closely coordinated experimental, computational, and theoretical efforts. The atomistic simulations have the unique ability to address highly nonequilibrium dynamic processes but suffer from the time- and length-scale limitations. A “mosaic approach” illustrated in Fig. 1, where the simulations performed at a range of laser fluences are mapped to different regions of the laser spot^16^, can partially alleviate the length-scale problem. The calculation of optical signatures of different states of the matter predicted in the atomistic simulations requires significant advances in the computational techniques based on the numerical solution of Maxwell equations^17^, as well as an improved theoretical understanding of optical properties of matter in the supercritical state and in the transient states of nanoscale phase separation. Experimentally, the combination of spatially-resolved optical scattering^7,18^ and reflectivity imaging^10–12^ with X-ray and electron diffraction probing^8,9^ can provide complementary information on different facets of the laser-induced phase explosion occurring under the confinement by a liquid environment. Overall, the synergy between the experimental and computational efforts appears to be a key factor in the successful exploration of the scientifically rich and practically relevant phenomenon of ultrashort pulse laser ablation in liquids.
